# Supplementary material for: The type IV secretion system of Patescibacteria is homologous to the bacterial monoderm conjugation machinery
Source: Microb Genom. 2025 May 23;11(5):001409. doi: 10.1099/mgen.0.001409 (PMC12102498; doi:10.1099/mgen.0.001409)
Supplement: Uncited Supplementary Material 1. [file mgen-11-01409-s001.pdf]

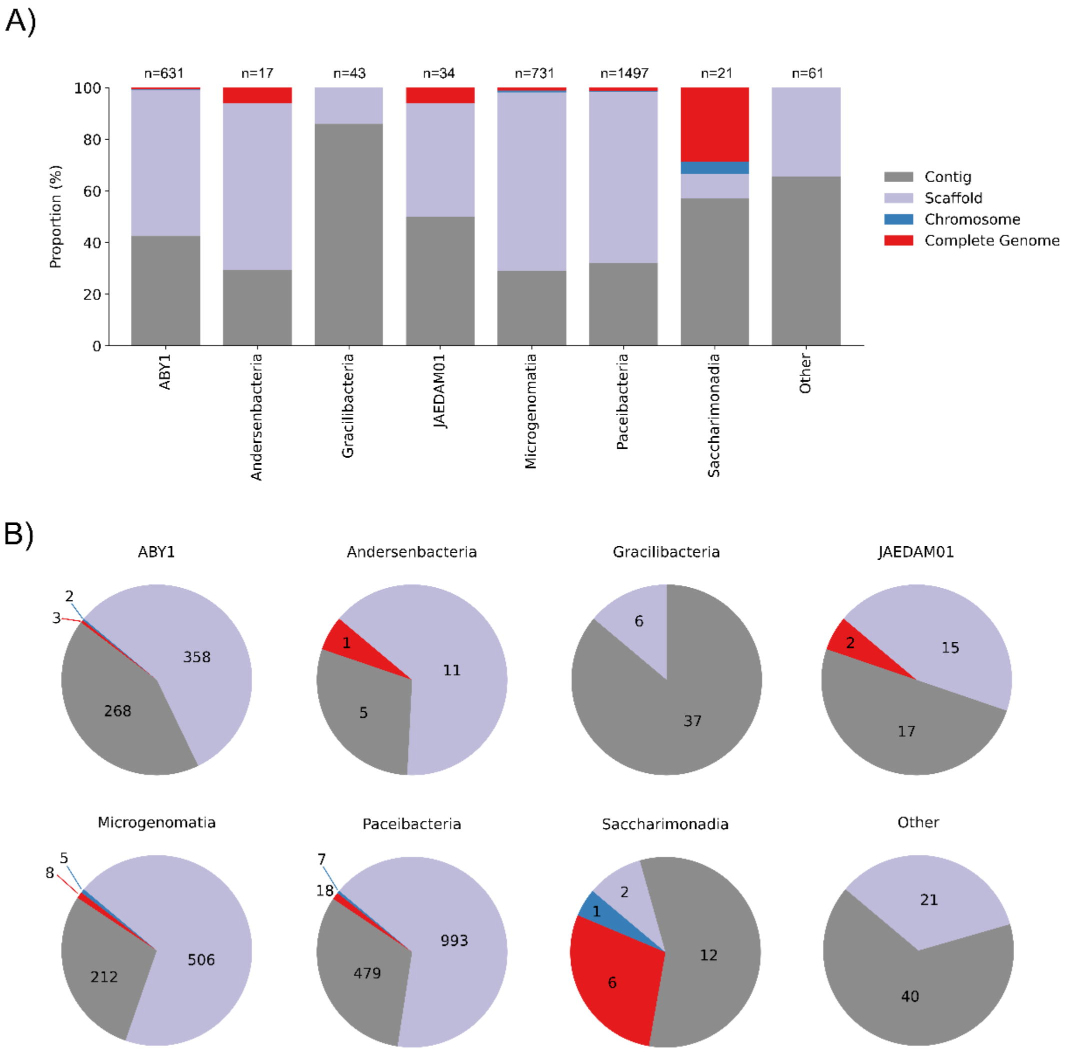

Supplementary Figure S1: Distribution of the *Pastescibacteria* dataset according to the assembly level. Different assembly levels for each *Patescibacteria* class are shown in various colors according to the legend, either as proportional percentages in stacked bars A) or as total counts in the pie chart B).

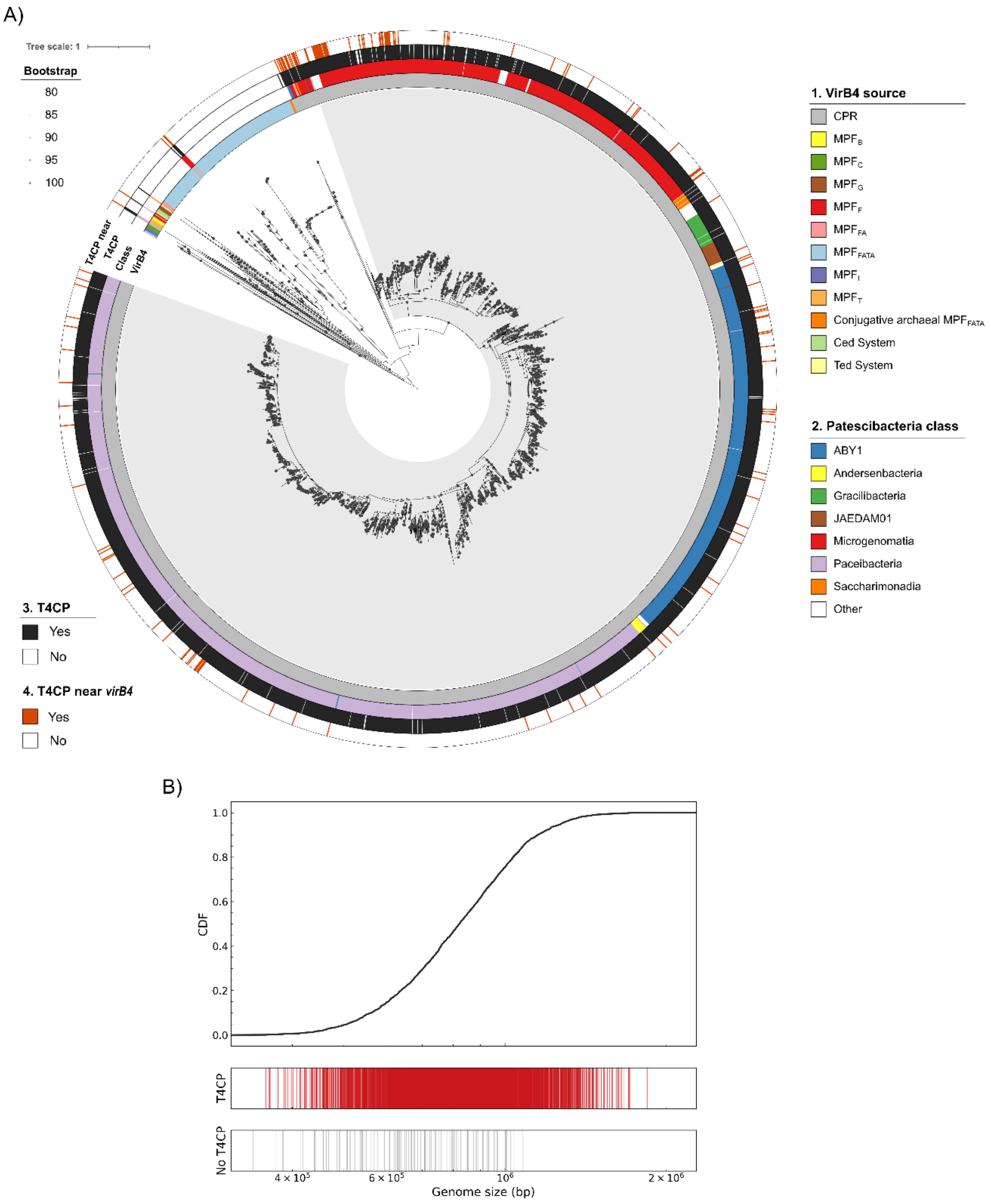

Supplementary Figure S2: Distribution of T4CP homologs in *Patescibacteria*. A) Maximum-likelihood tree of VirB4 proteins. The tree, along with rings 1 and 2, is as shown in Figure 2. Ring 3 displays the abundance of T4CP homologs, while ring 4 indicates those encoded near *virB4*. B) The *Patescibacteria* assemblies were ranked by size (307,478 – 2,280,175 bp), as shown on the x-axis. The top panel displays a cumulative distribution function (CDF) plot of genome size, while the lower panels use vertical lines to indicate the presence (red) or absence (gray) of a T4CP homolog in each corresponding genome.

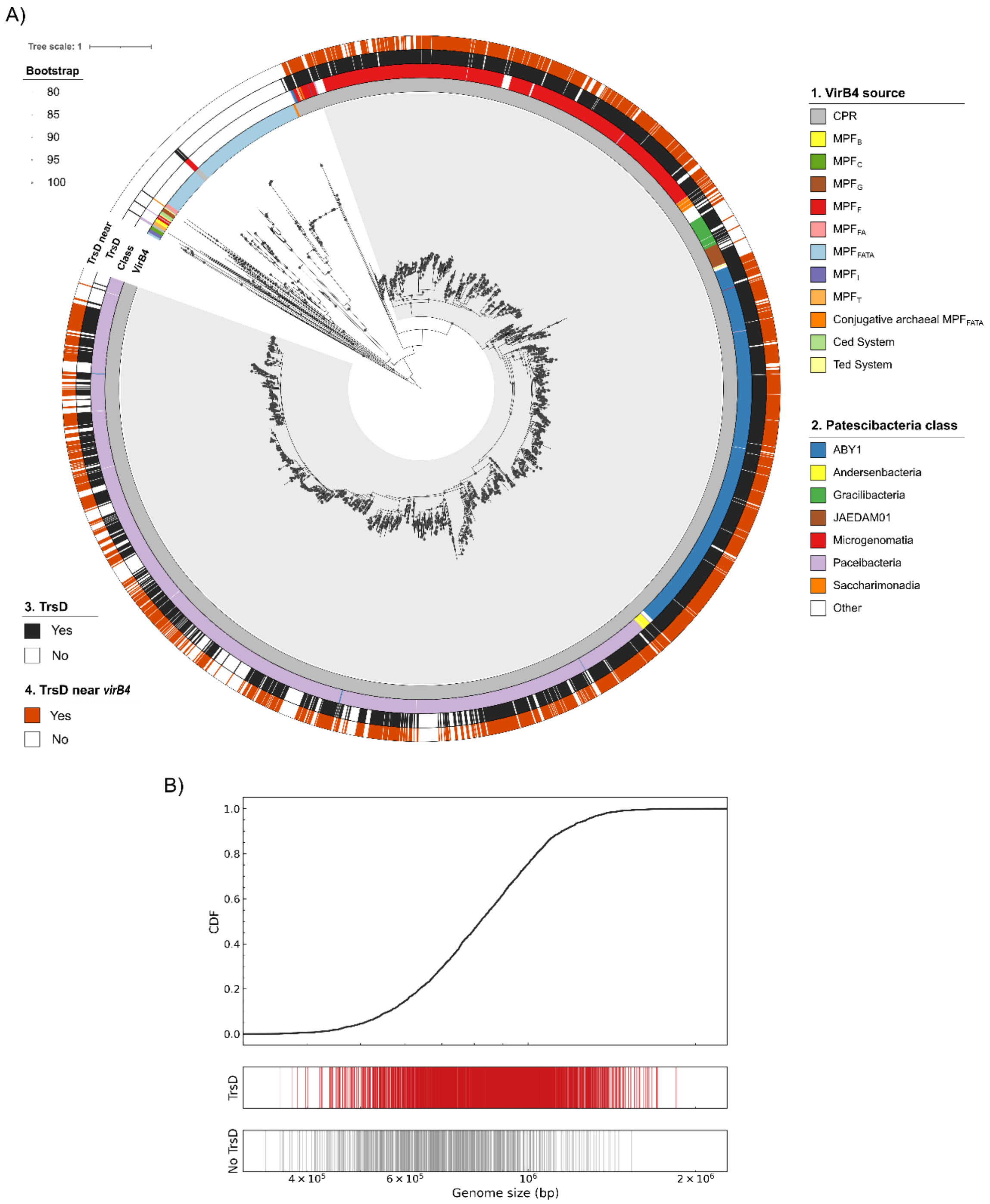

Supplementary Figure S3: Distribution of TrsD homologs in *Patescibacteria*. A) Maximum-likelihood tree of VirB4 proteins. The tree, along with rings 1 and 2, is as shown in Figure 2. Ring 3 displays the abundance of TrsD homologs, while ring 4 indicates those encoded near *virB4*. B) The *Patescibacteria* assemblies were ranked by size (307,478 – 2,280,175 bp), as shown on the x-axis. The top panel displays a cumulative distribution function (CDF) plot of genome size, while the lower panels use vertical lines to indicate the presence (red) or absence (gray) of a TrsD homolog in each corresponding genome.

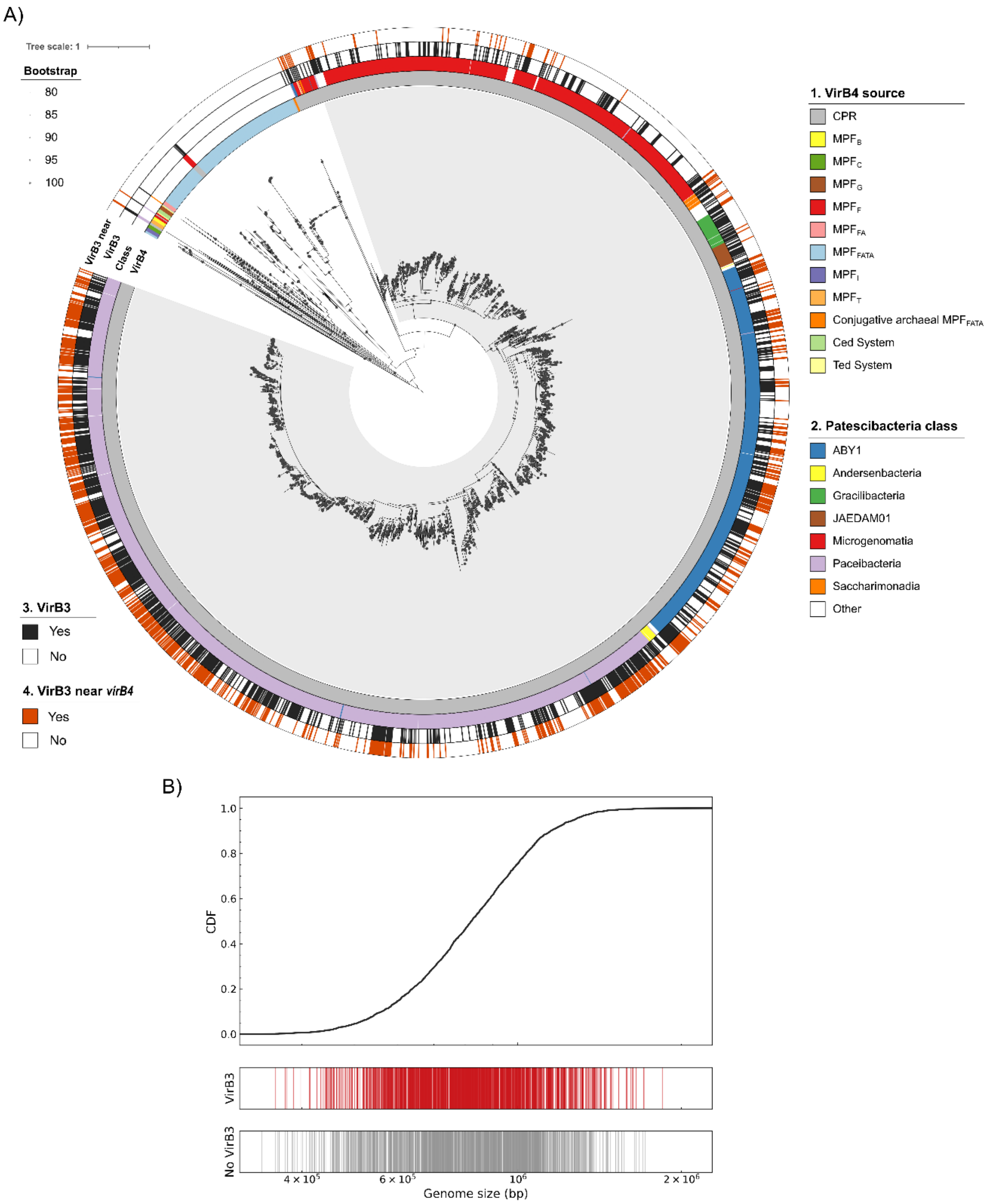

Supplementary Figure S4: Distribution of VirB3 homologs in *Patescibacteria*. A) Maximum-likelihood tree of VirB4 proteins. The tree, along with rings 1 and 2, is as shown in Figure 2. Ring 3 displays the abundance of VirB3 homologs, while ring 4 indicates those encoded near *virB4*. B) The *Patescibacteria* assemblies were ranked by size (307,478 – 2,280,175 bp), as shown on the x-axis. The top panel displays a cumulative distribution function (CDF) plot of genome size, while the lower panels use vertical lines to indicate the presence (red) or absence (gray) of a VirB3 homolog in each corresponding genome.

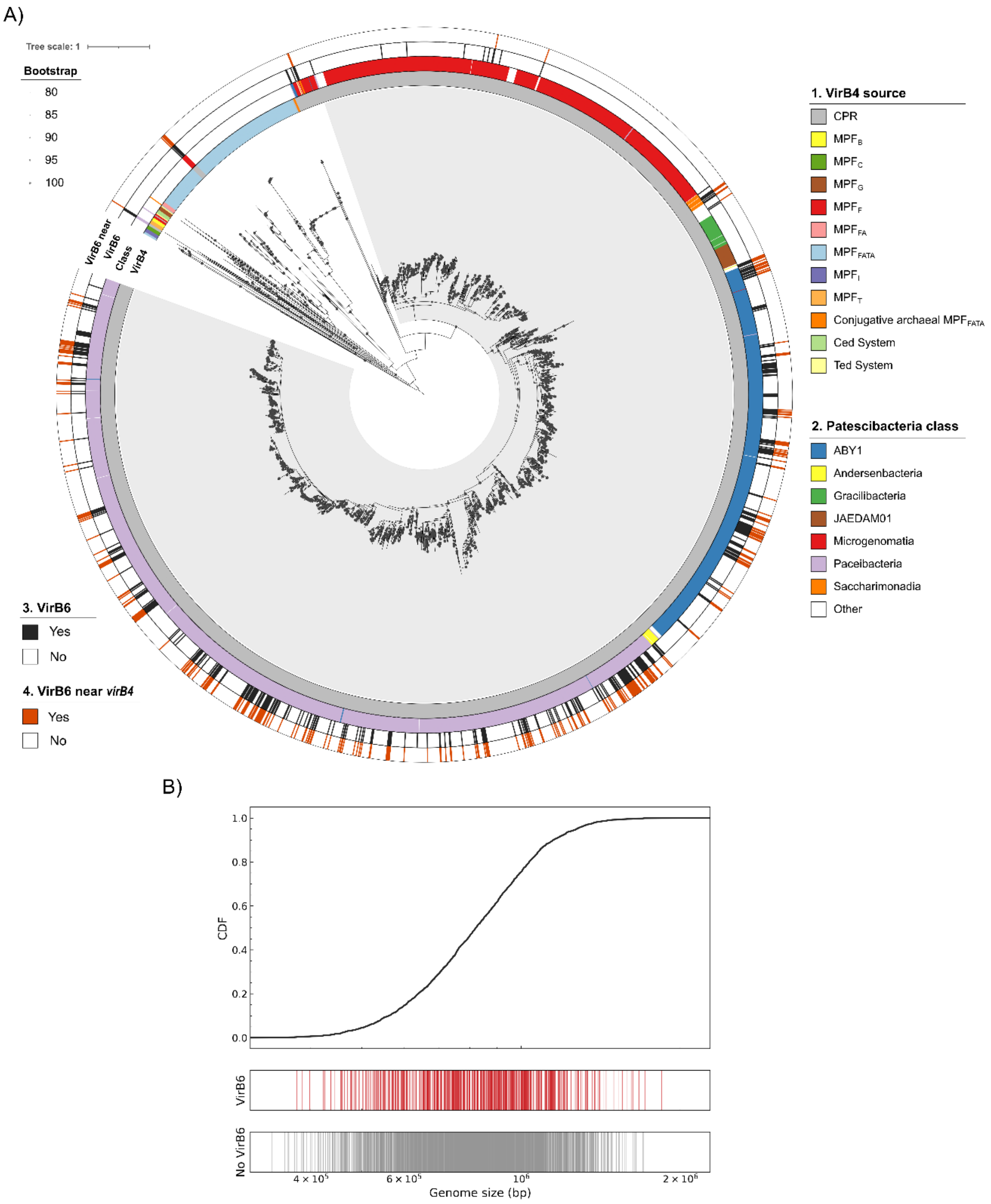

Supplementary Figure S5: Distribution of VirB6 homologs in *Patescibacteria*. A) Maximum-likelihood tree of VirB4 proteins. The tree, along with rings 1 and 2, is as shown in Figure 2. Ring 3 displays the abundance of VirB6 homologs, while ring 4 indicates those encoded near *virB4*. B) The *Patescibacteria* assemblies were ranked by size (307,478 – 2,280,175 bp), as shown on the x-axis. The top panel displays a cumulative distribution function (CDF) plot of genome size, while the lower panels use vertical lines to indicate the presence (red) or absence (gray) of a VirB6 homolog in each corresponding genome.

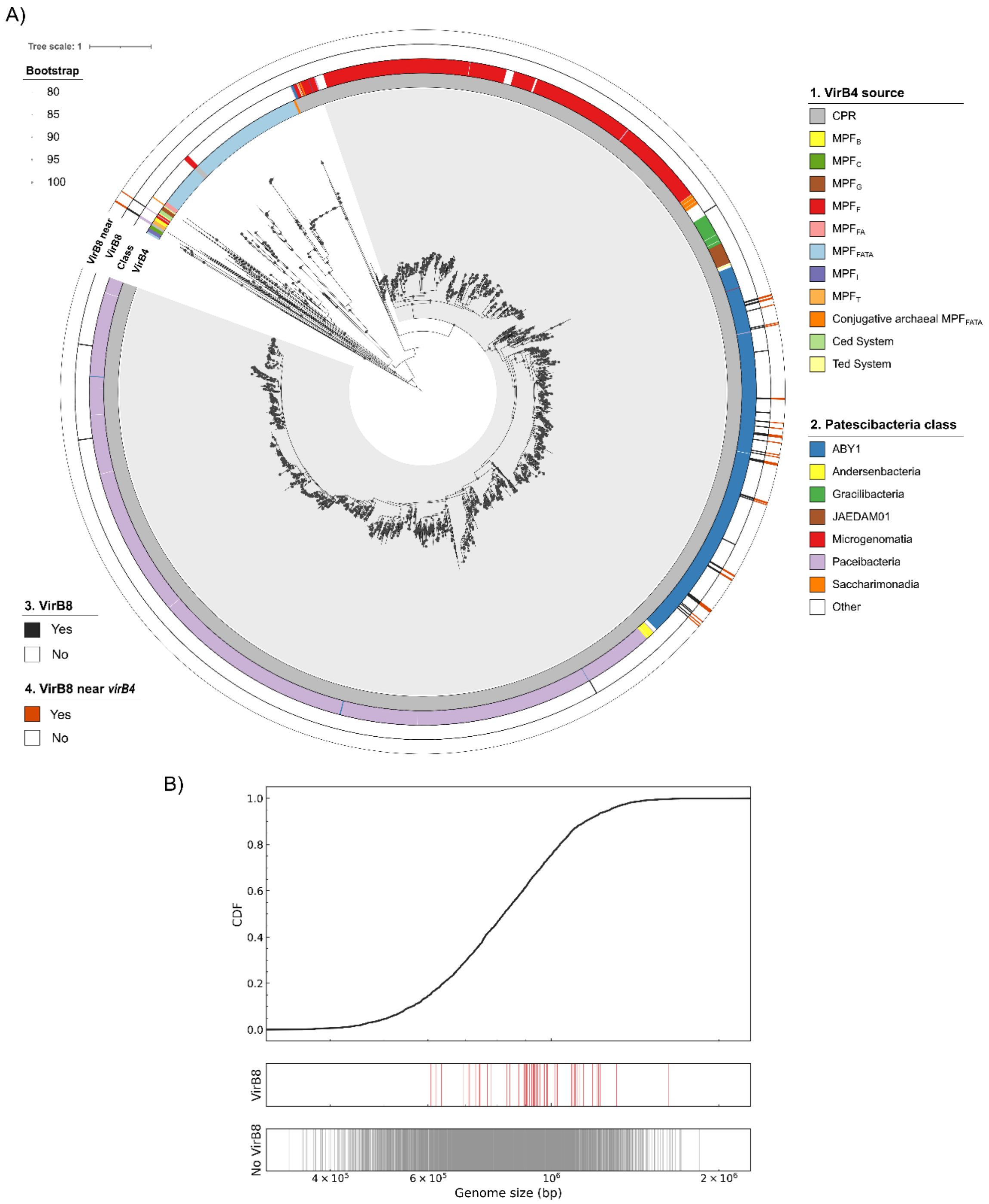

Supplementary Figure S6: Distribution of VirB8 homologs in *Patescibacteria*. A) Maximum-likelihood tree of VirB4 proteins. The tree, along with rings 1 and 2, is as shown in Figure 2. Ring 3 displays the abundance of VirB8 homologs, while ring 4 indicates those encoded near *virB4*. B) The *Patescibacteria* assemblies were ranked by size (307,478 – 2,280,175 bp), as shown on the x-axis. The top panel displays a cumulative distribution function (CDF) plot of genome size, while the lower panels use vertical lines to indicate the presence (red) or absence (gray) of a VirB8 homolog in each corresponding genome.

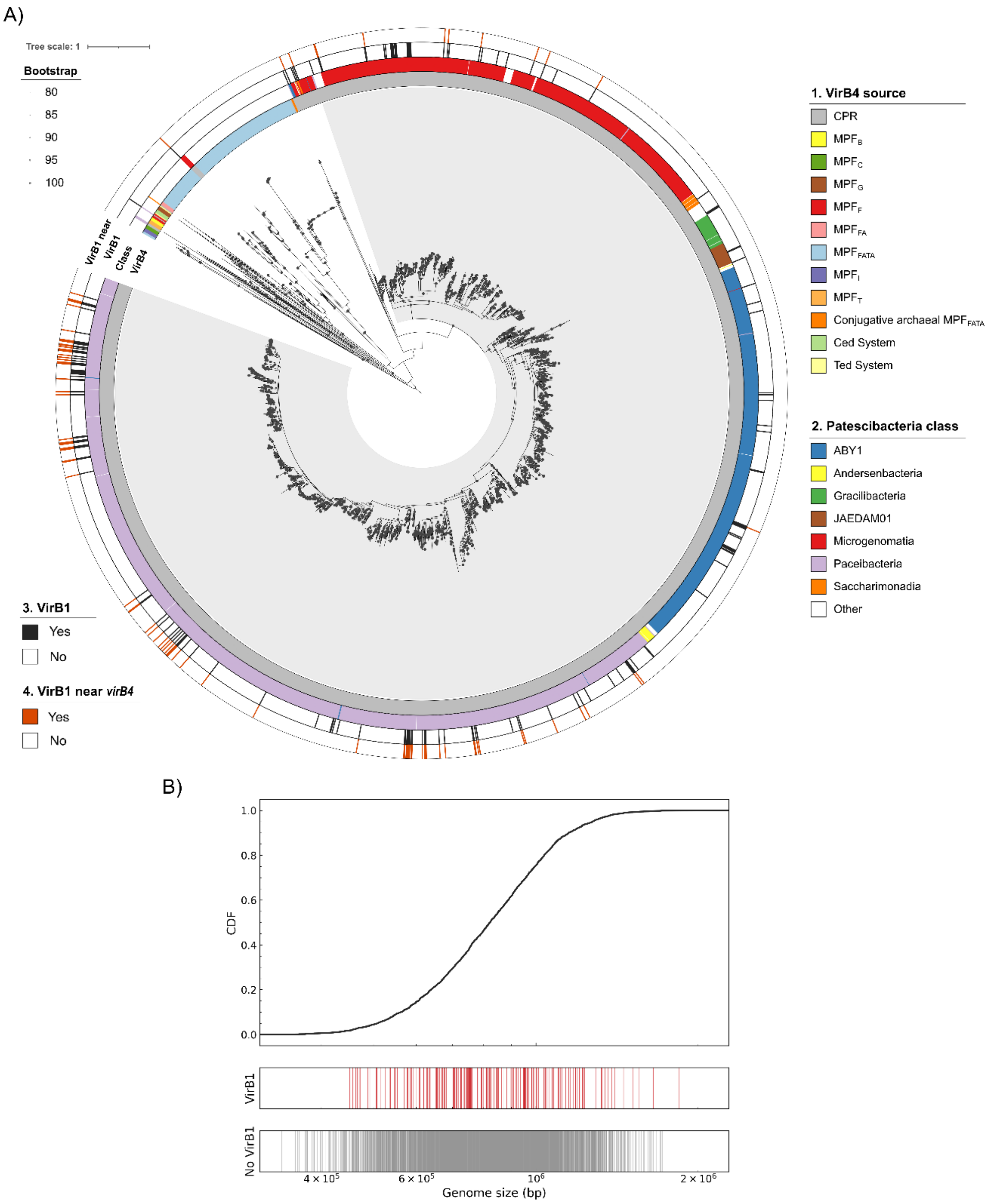

Supplementary Figure S7: Distribution of VirB1 homologs in *Patescibacteria*. A) Maximum-likelihood tree of VirB4 proteins. The tree, along with rings 1 and 2, is as shown in Figure 2. Ring 3 displays the abundance of VirB1 homologs, while ring 4 indicates those encoded near *virB4*. B) The *Patescibacteria* assemblies were ranked by size (307,478 – 2,280,175 bp), as shown on the x-axis. The top panel displays a cumulative distribution function (CDF) plot of genome size, while the lower panels use vertical lines to indicate the presence (red) or absence (gray) of a VirB1 homolog in each corresponding genome.

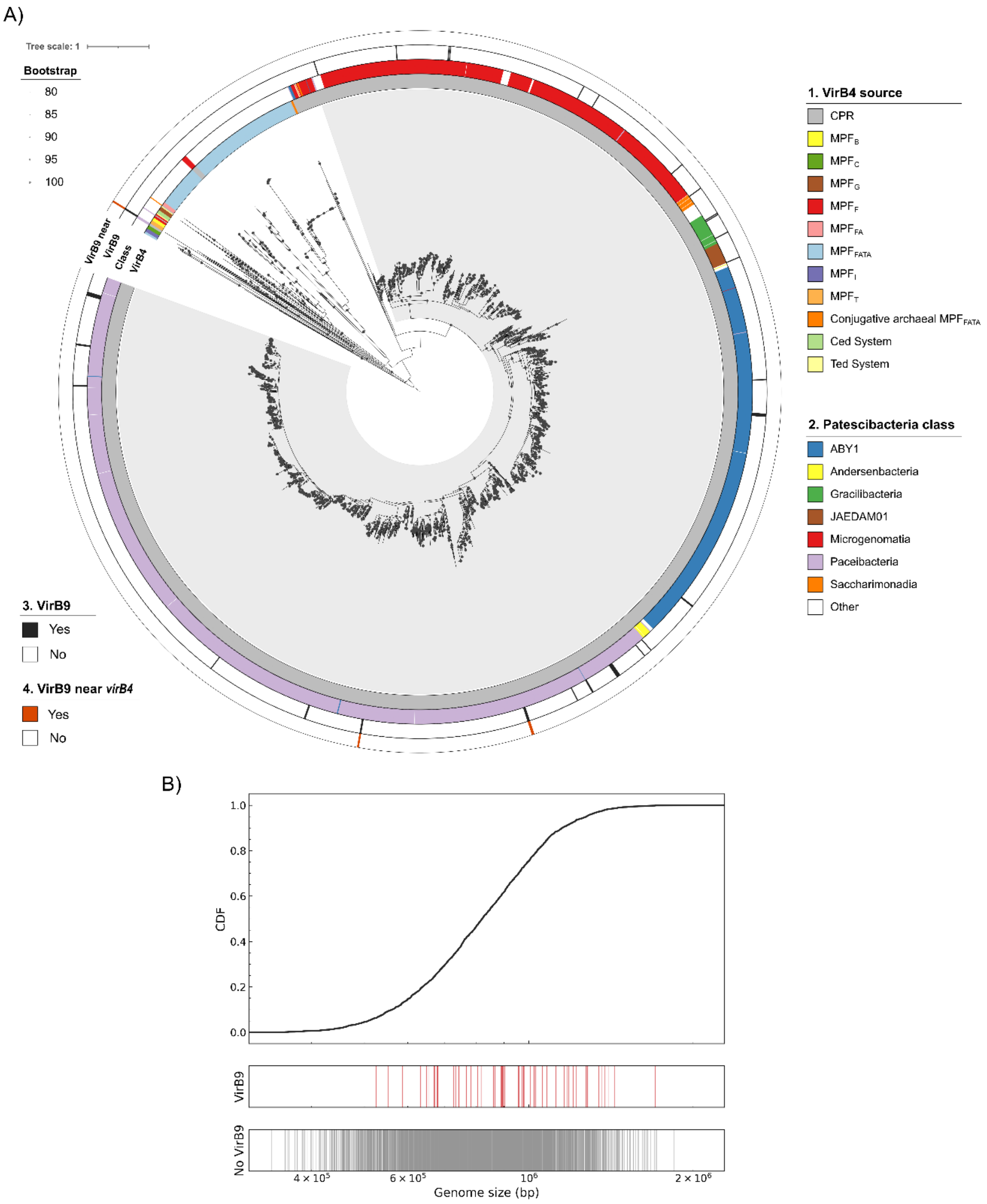

Supplementary Figure S8: Distribution of VirB9 homologs in *Patescibacteria*. A) Maximum-likelihood tree of VirB4 proteins. The tree, along with rings 1 and 2, is as shown in Figure 2. Ring 3 displays the abundance of VirB9 homologs, while ring 4 indicates those encoded near *virB4*. B) The *Patescibacteria* assemblies were ranked by size (307,478 – 2,280,175 bp), as shown on the x-axis. The top panel displays a cumulative distribution function (CDF) plot of genome size, while the lower panels use vertical lines to indicate the presence (red) or absence (gray) of a VirB9 homolog in each corresponding genome.

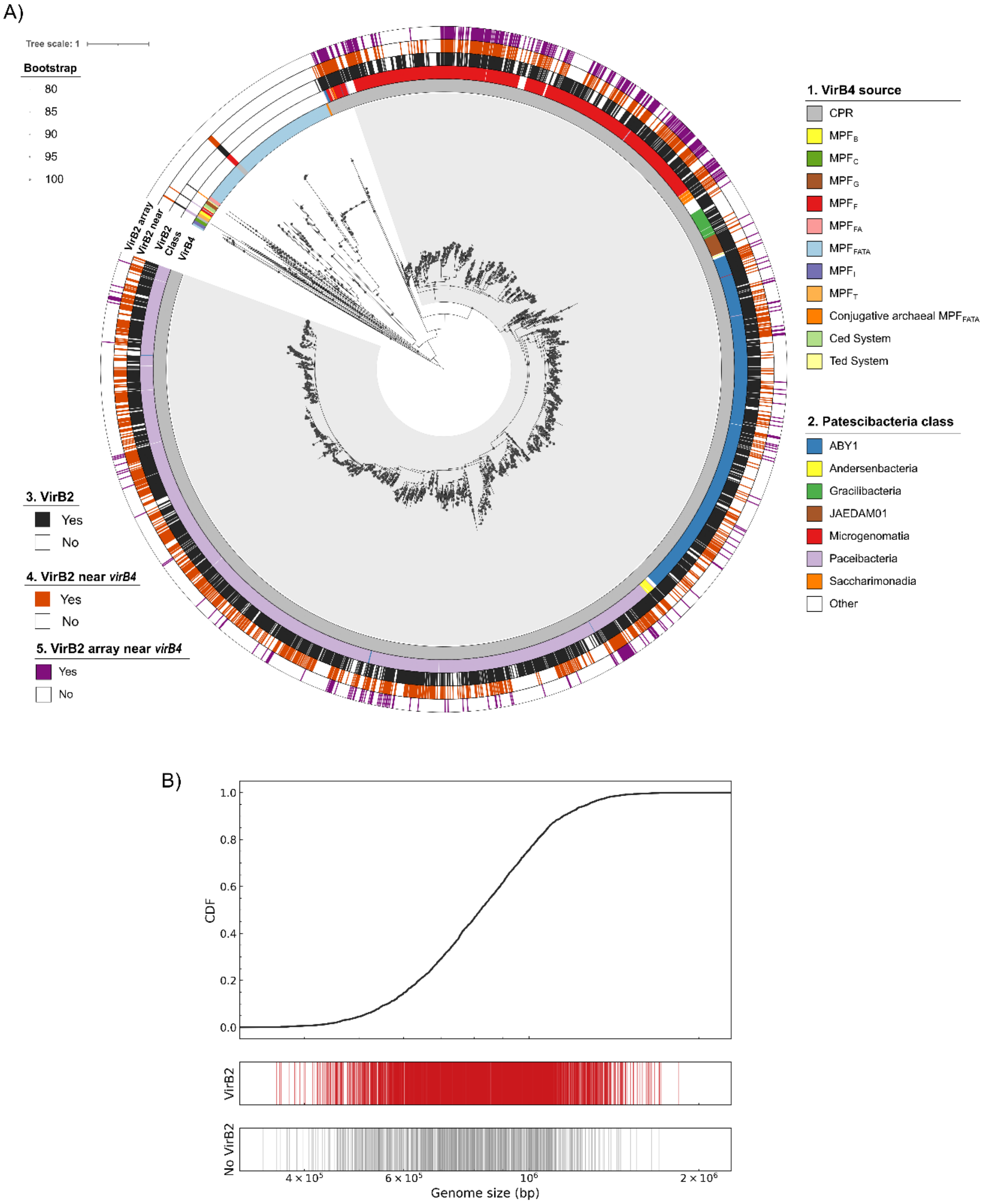

Supplementary Figure S9: Distribution of VirB2 homologs in *Patescibacteria*. A) Maximum-likelihood tree of VirB4 proteins. The tree, along with rings 1 and 2, is as shown in Figure 2. Ring 3 displays the abundance of VirB2 homologs, ring 4 indicates those encoded near *virB4* and ring 5 shows the presence of a VirB2 array near *virB4*. B) The *Patescibacteria* assemblies were ranked by size (307,478 – 2,280,175 bp), as shown on the x-axis. The top panel displays a cumulative distribution function (CDF) plot of genome size, while the lower panels use vertical lines to indicate the presence (red) or absence (gray) of a VirB2 homolog in each corresponding genome.

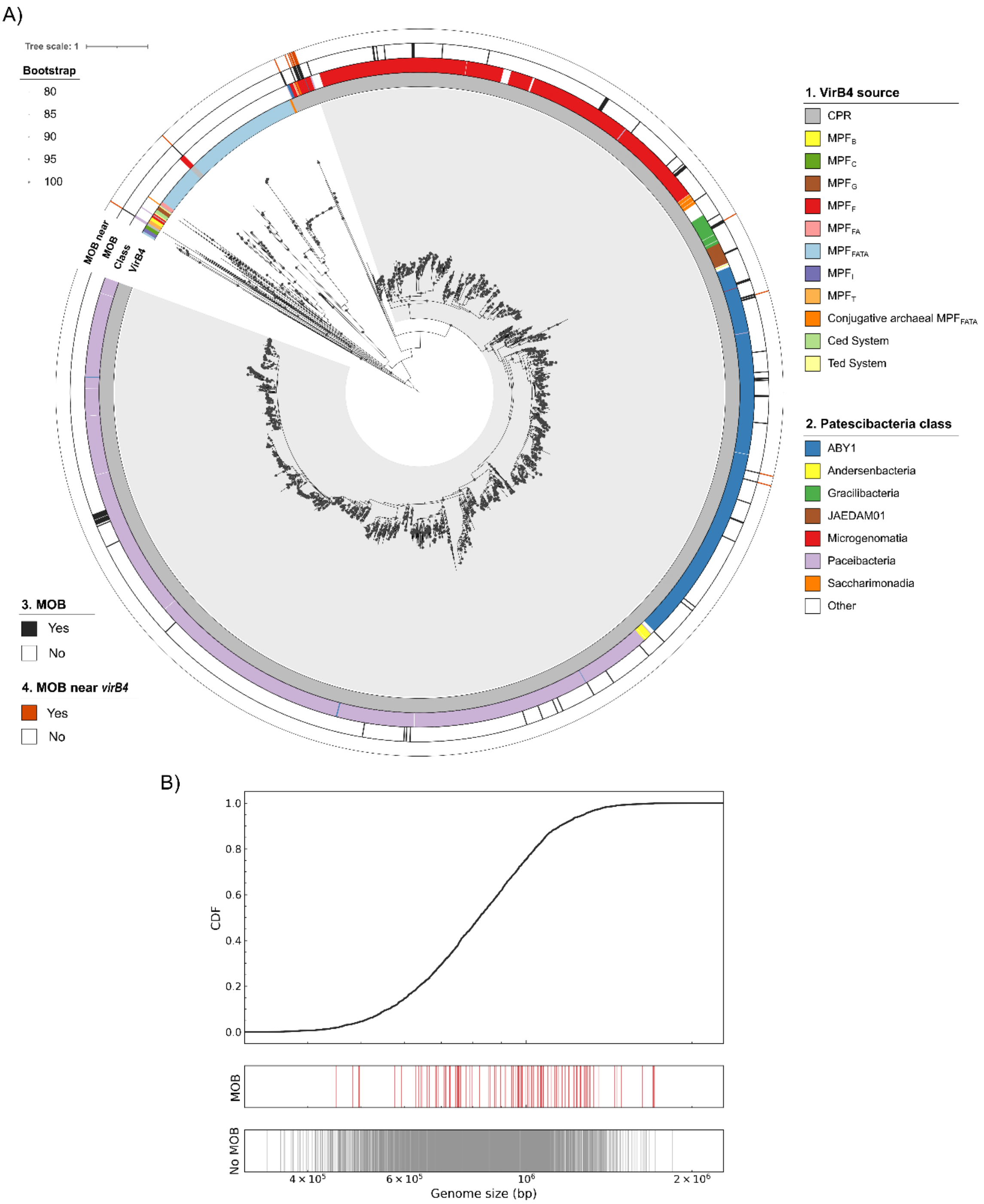

Supplementary Figure S10: Distribution of MOB relaxase homologs in *Patescibacteria*. A) Maximum-likelihood tree of VirB4 proteins. The tree, along with rings 1 and 2, is as shown in Figure 2. Ring 3 displays the abundance of MOB relaxase homologs, while ring 4 indicates those encoded near *virB4*. B) The *Patescibacteria* assemblies were ranked by size (307,478 – 2,280,175 bp), as shown on the x-axis. The top panel displays a cumulative distribution function (CDF) plot of genome size, while the lower panels use vertical lines to indicate the presence (red) or absence (gray) of a MOB relaxase homolog in each corresponding genome.
